# World hepatitis day in Burkina Faso, 2017: seroprevalence and vaccination against hepatitis B virus to achieve the 2030 elimination goal

**DOI:** 10.1186/s12985-018-1032-5

**Published:** 2018-08-06

**Authors:** Birama Diarra, Albert Theophane Yonli, Abdoul Karim Ouattara, Theodora Mahoukèdè Zohoncon, Lassina Traore, Christelle Nadembega, Dorcas Obiri-Yeboah, Justine Yara, Virginio Pietra, Paul Ouedraogo, Alain Bougouma, Rokia Sanogo, Jacques Simpore

**Affiliations:** 1 Biomolecular Research Center Pietro Annigoni (CERBA), BP 364, Ouagadougou 01, Burkina Faso; 2 Laboratory of Molecular Biology and Molecular Genetics (LaBioGene) UFR/SVT, University Ouaga I Prof Joseph KI-ZERBO, BP 7021, Ouagadougou 03, Burkina Faso; 3 Department of Microbiology and Immunology, School of Medical Sciences, University of Cape Coast, Cape Coast, Ghana; 4 SOS Hepatitis, Ouagadougou, Burkina Faso; 5 Saint Camille Hospital of Ouagadougou (HOSCO), BP 444, Ouagadougou 01, Burkina Faso; 6 Gastroenterology Service, CHU Yalgado OUEDRAOGO, Ouagadougou, Burkina Faso; 7 Faculty of Pharmacy, University of Technical Sciences and Technologies of Bamako (USTTB), Bamako, Mali

**Keywords:** Hepatitis B, Awareness, Screening, Vaccination

## Abstract

**Background:**

Burkina Faso is a high endemicity country for HBV infection. However, there are few data on vaccine coverage against HBV. The aim of this study was to contribute to the improvement of HBV vaccine coverage in Ouagadougou through HBV screening.

**Methods:**

Awareness campaigns and voluntary hepatitis B screening were organized in the twelve districts of Ouagadougou by the “SOS Hepatitis Burkina” association. A rapid HBsAg detection test (Abon Biopharma Guangzhou, Co., Ltd. Chine) was performed on 2216 individuals, who voluntarily answered a series of questions. Vaccination against hepatitis B was proposed to HBV negative participants.

**Results:**

In a sample of 2216 participants, aged 1 to 78 years (mean age 29.7 ± 14.7 years); a prevalence of 10.4% (230/2216) of HBsAg was obtained. This prevalence was high in the age groups 31 to 40 years (14.5%) and 41 to 50 years (15.0%). The prevalence of HBV was higher in the sixth district (14.3%) of Ouagadougou. At the end of the screening, 1202/1986 HBV negative participants were vaccinated, resulting in a vaccination rate of 60.5%. Vaccination coverage ranged from 44.5 to 73.7% all twelve districts.

**Conclusions:**

This study still reports a high prevalence of HBV infection among young people with a peak in the sixth district of Ouagadougou. The study achieved high vaccination coverage in all age groups and districts of Ouagadougou.

**Trial registration:**

The present study has been approved by the Ethics Committee for Health Research of Burkina Faso. CERS201501006 Registered 14 January 2015.

## Background

Hepatitis B, caused by the hepatitis B virus (HBV), is a major cause of liver disease morbidity and mortality worldwide [1]. Chronic HBV infection can cause liver cirrhosis and hepatocellular carcinoma (HCC) [2]. The World Health Organization (WHO) estimates an about two billion people are infected with this virus around the world and more than 360 million cases of chronic infections. About 686,000 deaths each year are attributed to the consequences of HBV infection [1]. HBV is transmitted parenterally via apparent percutaneous or per-mucous exposure to blood or other infected body fluids [3]. HBV infection is endemic in sub-Saharan Africa and causes about 44% of liver cirrhosis cases and 47% of hepatocellular carcinoma cases [4].

Burkina Faso is a country with high HBV endemicity with prevalence of more than 8% in the general population [5]. Prevalence of 9.5 to 14.5% of HBV infection has been reported in the general population of Ouagadougou [6, 7]. In addition, prevalence rates of 12.1 and 9.3% were reported among the staff of Nanoro health district and pregnant women in Ouagadougou, respectively [8, 9]. It varied between 13 and 19% among blood donors in Burkina Faso [6, 10, 11].

The risk of acquiring HBV infection in Burkina Faso remains high, not only because of its high prevalence, but also because of the low vaccination rate among the general population. Prevention seems to be the public health intervention with the greatest impact on the elimination of viral hepatitis [12]. It is within this framework that the World Health Organization (WHO) has announced its strategy prioritizing actions to eliminate viral hepatitis B in most parts of the world and control it in other regions by 2030 [13]. Thus, it has set targets to be achieved by 2030, namely: a 90% reduction in new cases of chronic hepatitis B and C and a 65% reduction in mortality due to these two infections [14].

Many countries in sub-Saharan Africa are now in the process of developing viral hepatitis management guidelines and strategic plans to achieve these goals for viral hepatitis elimination [15]. In Burkina Faso, the lack of better organization of awareness campaigns, screening and vaccination against hepatitis B remain one of the obstacles to HBV elimination. Thus, a study carried in Burkina Faso showed that development of awareness and screening campaigns on HBV can increase the vaccination coverage in the population [7]. Therefore, the objective of this study was to contribute to the improvement vaccine coverage against hepatitis B in the population of Ouagadougou through HBV screening and vaccination.

## Methods

### Study population

The present study was approved by the Ethics Committee for Health Research of Burkina Faso (DELIBERATION - N° 2015–01-006) and the Institutional Ethics Committee of the Center for Biomolecular Research Pietro Annigoni/Laboratory of Molecular Biology and Genetics (CERBA/LaBioGene) [7]. The present study was conducted in Ouagadougou, Burkina Faso in July 2017 on World Hepatitis Day. The study was carried out in collaboration with the non-profit “SOS Hépatite Burkina” association during the awareness and screening campaign, which took place during July 10–30, 2017. The campaign was implemented in the twelve districts of Ouagadougou city. We used media (radio and television), advertisements, brochures, and conferences to raise awareness. Awareness focused on the routes of transmission, risk groups, symptoms, complications, and prevention against hepatitis virus infection. Participation was free and voluntary, and participants answered a questionnaire on age, sex, marital status, profession, and vaccination status for HBV.

### Sample collection

The convenience sampling method was used in this study to select volunteers attending the campaign; the sampling process was preceded by individual counseling. All study participants gave their free written and informed consent according to the Helsinki Declarations during this pretest counseling and all volunteers were included in the study, regardless of age, gender, profession, or social status. People who were known to be HBsAg-positive already were excluded from the study. Venous blood samples, collected from HBsAg-positive subjects, were centrifuged, and the plasma was stored at − 20 °C until further use.

### Serology of HBsAg

The HBV rapid test kit (ABON Biopharm Hangzhou Co. Ltd., China; sensitivity > 99.0%; specificity, 96.8%; accuracy, 98.3%) [16, 17] was used for the rapid diagnosis of HBsAg. The results were presented to the participants during individual posttest counseling. Vaccination with a recombinant hepatitis B vaccine (EUVAX-B®) was recommended to HBV-negative participants, based on the absence of HBV vaccination history (AbHBc and AbHBs). “SOS Hepatitis Burkina” association is in close collaboration with general practitioners and gastroenterologists for follow-up and adequate management of study participants tested positive for hepatitis B and vaccination of HBsAg-negative individuals throughout the year. However, the latter should contribute (32 Euros for the 3 doses) for vaccination against hepatitis B because of lack of funding for vaccination in this study. On-site vaccination was provided by the association during the study period and at its headquarters throughout the year, thus making it possible to track the vaccination rates. When they received the first dose of the vaccine during the study period, participants were then follow-up by a text message reminder for the next vaccine dose, within the week of its due date until the completion of the vaccine three doses.

#### Limit of the study

Due to limited financial resources and lack of vaccine subsidies, we could not perform HCV screening for all the participants and were unable to vaccinate all HBsAg-negative subjects. Vaccinated cases received the first dose of HBV vaccine after screening. The second and third doses were administered at 1 and 5 months after the second dose, respectively. Except the first dose of the hepatitis B vaccine, the second and third doses were administered by appointment at the headquarters of the “SOS Hepatitis Burkina” association.

### Statistical analysis

The data collected was analyzed using SPSS 21.0 and Epi info version 7.0 software. The chi-square test was used for comparisons, and *p* value ≤0.05 was considered statistically significant.

## Results

### Sociodemographic characteristics of the study population

Table 1 shows the sociodemographic characteristics of the study population. Two thousand two hundred sixteen (2216) participants, including 1221 (55.1%) women and 995 (44.9%) men, aged 1 and 78 years (mean age 29.7 ± 14.7 years), participated in this screening campaign.

**Table 1:** Sociodemographic Characteristics of the Study Population (*n* = 2216)

Variables	N	%	Mean age
Sex			
Female	1221	55.1	29.6 ± 14.5
Male	995	44.9	29.8 ± 15.0
Age			
≤ 20	545	24.6	11.6 ± 5.6
21–30	719	32.4	26.0 ± 2.8
31–40	516	23.3	35.0 ± 2.8
41–50	207	9.3	45.1 ± 2.7
> 50	229	10.3	58.5 ± 7.5
Activity			
Trader	164	7.4	36.3 ± 12.1
Pupil/Student	843	38.0	16.6 ± 8.7
Civil servant	530	24.0	40.5 ± 11.4
Housewife	278	12.5	41.5 ± 11.9
Informal sector	401	18.1	36.0 ± 11.3
Marital Status			
Single	1290	58.2	20.8 ± 10.8
Divorced	13	0.6	45.0 ± 10.0
Married	886	40.0	41.0 ± 11.5
Widow	27	1.2	51.8 ± 8.6
Serology (HBsAg)			
Negative	1986	89.6	29.2 ± 15.0
Positive	230	10.4	33.8 ± 11.6

Young people constitute the majority age groups (≤ 20 years is 24.6%, and that 21 to 30 years is 32.4%) (Table 1). Depending on the type of activity, the majority were students (38.0% or 843/2216), followed by civil servants (24.0% or 530/2216). In terms of marital status, 58.2% (1290/2216) were single, and 40.0% (886/2216) were married (Table 1). Among 2216 screened cases, 230 (10.4%) were HBsAg-positive (Table 1).

### HBV infection by sociodemographic characteristics of study population (*N* = 2216)

The prevalence of HBV was higher among men (13.4%) than women (7.8%) with a statistically significant difference (*p* = 0.001) (Table 2). It was high in the age groups 31 to 40 years (12.5%) and 41 to 50 years (15.0%) (Table 2). HBV infection was higher among informal sector workers (14.4%), traders (12.8%) and civil servants (12.2%). It was lower among students (7.6%) (Table 2). About 11.6% of married people were HBV infected compared to 11.1% of single people.

**Table 2:** HBV infection by sociodemographic characteristics of study population (*N* = 2216)

Variables	HBV	%	*P* value
	Positive/Total		
Sex			
Female	96/1221	7.8	< 0.001
Male	134/995	13.4	
Age			
≤ 20	19/545	3.5	Ref
21–30	81/719	11.3	0.0001
31–40	75/516	14.5	0.0001
41–50	31/207	15.0	0.0001
> 50	24/229	10.5	0.0001
Activity			
Pupil/Student	64/843	7.6	Ref
Trader	21/164	12.8	0.0280
Civil servant	65/530	12.2	0.0038
Housewife	22/278	7.9	0.8612
Informal sector	58/401	14.4	0.0001
Marital status			
Single	150/1290	11.6	–
Widow and divorced	2/40	5.0	0.2958
Married	98/886	11.1	0.6826

### Vaccination rate following HBV screening by sex and age group

Vaccination against hepatitis B was recommended to HBV-negative participants. Among 1986 HBV-negative subjects in this campaign, 1202 participants received all three doses of the vaccine giving a vaccination rate of 60.5% (Table 3). Among vaccinated persons, 55.6% (668/1202) were women and 44.4% (534/1202) were men.

**Table 3:** Vaccination rate following HBV screening by sex and age group

Variables	Vaccinated^a^/Total	%	1st dose	2nd dose	3rd dose	Mean age
Sex						
Female	668/1139	58.6	675	672	668	29.8 ± 13.8
Male	534/847	63.0	545	535	534	30.1 ± 14.1
Total	1202/1986	60.5	1220	1207	1202	24.6 ± 4.7
Age						
≤ 20	210/474	44.3	215	211	210	12.8 ± 5.4
21–30	436/664	65.7	441	437	436	26.1 ± 2.8
31–40	316/459	68.8	318	317	316	35.0 ± 2.8
41–50	119/192	62.0	122	120	119	45.0 ± 2.8
> 50	121/197	61.4	124	122	121	57.5 ± 6.0
Number of vaccinated individuals						
Female	668/1202	55.6	675	672	668	29.5 ± 12.7
Male	534/1202	44.4	545	535	534	30.1 ± 11.2
Vaccination rate						
Female	668/1986	33.6	675	672	668	29.8 ± 13.8
Male	534/1986	26.9	545	535	534	29.9 ± 13.9

^a^All vaccinated HBsAg individuals completed their three doses of hepatitis B vaccine

The vaccination rate was statistically higher among women than men (33.6% vs 26.9%, *p* < 0.001) (Table 3). The vaccinated persons were mostly young people whose mean age was 24.6 ± 4.7 years (Table 3).

### Distribution of HBV infection and vaccination rate according to the districts of Ouagadougou city

The prevalence of HBV infection ranged from 6.6% in districts 5 and 12, to 14.3% in district 6 (Table 4). Vaccination coverage in all twelve districts and ranged between 44.5 to 73.7% (Table 4). The prevalence of HBV infection was significantly higher in district six compared to district four (*p* = 0.024), five (*p* = 0.002) and twelve (*p* = 0.047). District 1 also had a significantly higher prevalence compared to district 5 (*p* = 0.035).

**Table 4:** Distribution of HBV infection and vaccination rate according to the districts of Ouagadougou city

Districts	HBV			Vaccination		
	N	Positive	%	N	Vaccinated	%
1*	214	26	12.1	188	136	72.3
2	111	12	10.8	99	73	73.7
3	245	23	9.4	222	140	63.1
4^*^	260	22	8.5	238	168	70.6
5^*^	259	17	6.6	242	173	71.5
6^*^	413	59	14.3	354	214	60.4
7	218	23	10.5	195	90	46.2
8 and 9	52	4	7.7	48	26	54.2
10	173	18	10.4	155	69	44.5
11	180	20	11.1	160	74	46.3
12^*^	91	6	6.6	85	39	45.9

^*^Comparison of the prevalence of HBV infection among different districts: 1 vs 5 (*p* = 0.035), 4 vs 6 (*p* = 0.024), 5 vs 6 (0.002), 6 vs 12 (*p* = 0.047)

## Discussion

Since 2010, WHO has instituted the World Hepatitis Day in order to enable people to better know this infection and ways to protect themselves. Thus, every year, on the occasion of this day, the “SOS Hepatitis” association conducts voluntary screening activities against hepatitis B in Ouagadougou city. This year, the overall seroprevalence of hepatitis B virus was 10.4%, including 6.1% for men and 4.3% for women. This prevalence of 10.4% is lower than 14.5% reported in the general population of Ouagadougou [18]; and those 12.9 to 17.3% reported among blood donors in Burkina Faso [19, 20]. However, our prevalence is similar to the 9.8% reported in the general population of Ouagadougou [7]; and 9.3 to 9.8% reported among pregnant women in Burkina Faso [5, 8].

The variations in prevalence rates could be explained by the size and type of the study population, by the limitations of serological tests use, immunity and other socioeconomic characteristics. It should be also mentioned that voluntary participation in a screening program introduces self-selection bias. The variation in the prevalence of HBV infection in the twelve districts of Ouagadougou (4.2 to 14.3%) could be explained by the difference in population participation rates to voluntary screening campaigns. HBV infection has a distribution of leopard spots with areas of high prevalence (district 1 and 6) corresponding to black spots separated by areas of low prevalence (district 4, 5 and 12). These results demonstrate that the frequency of HBV infection varies not only across countries, but also between different geographical areas within a city and suggest that HBV infection prevalence data are needed at sub-national level to estimate disease burden and guide health and vaccine policy. These differences in HBV infection prevalence between cities could be due to a lack of infrastructure within certain cities to provide hepatitis B vaccine especially since the best-developed areas had the lowest prevalence in this study.

The high prevalence of 11.3 and 14.5% found in the 21 to 30 and 31 to 40 age groups in this study, respectively, show that young people are the most affected by HBV infection. These young people constituted also the most important group of volunteer screening. These results corroborate those of 15.9 and 16.3% of Tao et al. [18] and 11.6 and 13.1% of Diarra et al. [7] reported among young people in Ouagadougou, Burkina Faso. They are also similar to those of 22.2% of Makuwa et al. [21] reported among young people in Gabon. This high prevalence of HBV among young people could be explained by use of nonsterile syringes among intravenous drug users, customary scarification rites, male and female circumcision with soiled blades. The reduction in prevalence from 15.0 to 10.5% observed in the age groups 41 to 50 and > 50 years, respectively, could be due to the increase in the number of deaths following liver cirrhosis or hepatocellular carcinoma in patients > 50 years.

Despite the consequences that may be caused by HBV infection, there is no national strategy or plan for awareness and to fight against viral hepatitis B in Burkina Faso, because viral hepatitis B is not considered a priority disease. However, the present study achieved a 60.5% (1202/1986) vaccine coverage against hepatitis B after screening. This vaccination rate evolved around 50.0% in all age groups and between 44.5 and 73.7% in all districts of Ouagadougou city. The high participation rate (95.0% or 1887/1986) to vaccination is an affirmation of the Burkinabe people’s concern about viral hepatitis B.

However, in absence of a subsidy, the cost of the vaccine remains high and constitutes a barrier despite the willingness of participants to be vaccinated against hepatitis B. A recent study reports that to achieve the WHO goals of eliminating HBV by 2030, sub-Saharan Africa will need to actively prioritize implementation of several elimination strategies.

“In addition, antenatal HBsAg screening, prevention of mother-to-child transmission with administration of tenofovir to pregnant women who are HBV monoinfected with HBV DNA concentrations higher than 20,000 IU/mL, together with HBV birth-dose vaccination, and ensuring subsequent full HBV-vaccine coverage should form the cornerstone of any HBV elimination strategy [15]”.^1^ Chotun et al. [22] also reported the importance of HBV screening among pregnant women and the need to vaccinate newborns within 24 h of birth. We hope that the report will also stimulate dialogue with respect to the roles of all stakeholders in forging a cohesive and effective response to viral hepatitis in Burkina Faso. Thus, we suggest that the government considers viral hepatitis B a priority disease, commemorate World Hepatitis Day and promote other awareness raising activities.

## Conclusion

The voluntary screening campaign of the non-profit “SOS Hépatite Burkina” association in 2017 reported a prevalence of 10.4% of HBsAg in the population of Ouagadougou. Prevalence was relatively high among young people and in the majority of districts of Ouagadougou city. This campaign achieved a vaccination coverage of 60.5% after screening. This coverage was high in all age groups and in all districts of Ouagadougou city. The study suggests an active involvement of the Burkinabe government in the fight against viral hepatitis B through its recognition as a priority disease, commemorate World Hepatitis Day and promote other awareness raising activities and the reduction of cost of the vaccine.

## Abbreviations

CERBA: Pietro Annigoni Biomolecular Research Center
HBsAg: Hepatitis B Surface Antigen
HBV: Hepatitis B Virus
HCC: Hepatocellular Carcinoma
LaBioGene: Laboratory of Molecular Biology and Genetics
WHO: World Health Organization

## Footnotes

1 Spearman et al. [15] Page 907.

## References

[1] WHO. Global hepatitis report 2017. World Health Organization Geneva; 2017. http://www.who.int/hepatitis/publications/global-hepatitis-report2017/en/. Accessed 02 Aug 2018.

[2] Zhou JY, Zhang L, Li L, Gu GY, Zhou YH, Chen JH (2012). High hepatitis B virus load is associated with hepatocellular carcinomas development in Chinese chronic hepatitis B patients: a case control study. Virol J.

[3] Chien YC, Jan CF, Kuo HS, Chen CJ (2006). Nationwide hepatitis B vaccination program in Taiwan: effectiveness in the 20 years after it was launched. Epidemiol Rev.

[4] Perz JF, Armstrong GL, Farrington LA, Hutin YJ, Bell BP (2006). The contributions of hepatitis B virus and hepatitis C virus infections to cirrhosis and primary liver cancer worldwide. J Hepatol.

[5] Simpore J, Savadogo A, Ilboudo D, Nadambega MC, Esposito M, Yara J (2006). Toxoplasma gondii, HCV, and HBV seroprevalence and co-infection among HIV-positive and -negative pregnant women in Burkina Faso. J Med Virol.

[6] Tao I, Compaoré TR, Diarra B, Djigma F, Zohoncon TM, Assih M (2014). Seroepidemiology of hepatitis B and C viruses in the general population of Burkina Faso. Hepatitis research and treatment..

[7] Diarra B, Ouattara AK, Wendkuuni Djigma F, Rebeca Compaore T, Obiri-Yeboah D, Traore L, Theophile Soubeiga S, Bado P, Yara J, Pietra V (2017). World Hepatitis Day in Burkina Faso, 2016: Awareness, Screening, Identification of HBV Markers, HBV/HCV Coinfection, and Vaccination. Hepat Mon..

[8] Simpore J, Granato M, Santarelli R, Nsme RA, Coluzzi M, Pietra V (2004). Prevalence of infection by HHV-8, HIV, HCV and HBV among pregnant women in Burkina Faso. J Clin Virol.

[9] Pietra V, Kiema D, Sorgho D, Kabore S-PCG, Mande S, Castelli F (2008). Prévalence des marqueurs du virus de l’hépatite B et des anticorps contre le virus de l’hépatite C parmi le personnel du District Sanitaire de Nanoro, Burkina Faso. Science et technique, Sciences de la santé.

[10] Nagalo MB, Sanou M, Bisseye C, Kabore MI, Nebie YK, Kienou K (2011). Seroprevalence of human immunodeficiency virus, hepatitis B and C viruses and syphilis among blood donors in Koudougou (Burkina Faso) in 2009. Blood Transfus.

[11] Nagalo BM, Bisseye C, Sanou M, Kienou K, Nebie YK, Kiba A (2012). Seroprevalence and incidence of transfusion-transmitted infectious diseases among blood donors from regional blood transfusion centres in Burkina Faso. West Africa Trop Med Int Health.

[12] Brahm J, Castera L, Hou J, Lindor K (2016). Joint society statement for the elimination of viral hepatitis. Hepatol.

[13] Towards elimination of viral hepatitis by 2030. Lancet. 2016;388:308. 10.1016/S0140-6736(16)31144-8 27477148

[14] WHO (2016). Global health sector strategy on viral hepatitis 2016–2021: towards ending viral hepatitis.

[15] Spearman CW, Afihene M, Ally R, Apica B, Awuku Y, Cunha L (2017). Hepatitis B in sub-Saharan Africa: strategies to achieve the 2030 elimination targets. Lancet Gastroenterol Hepatol.

[16] Chizzali-Bonfadin C, Adlassnig KP, Kreihsl M, Hatvan A, Horak W (1997). Knowledge-based interpretation of serologic tests for hepatitis on the world wide web. Clin Perform Qual Health Care.

[17] Ter Borg F, Ten Kate FJW, Cuypers HTM, Leentvaar-Kuijpers A, Oosting J, Wertheim-van Dillen PME (1998). Relation between laboratory test results and histological hepatitis activity in individuals positive for hepatitis B surface antigen and antibodies to hepatitis B e antigen. Lancet.

[18] Tao I, Compaore TR, Diarra B, Djigma F, Zohoncon TM, Assih M (2014). Seroepidemiology of hepatitis B and C viruses in the general population of Burkina Faso. Hepat Res Treat.

[19] Collenberg E, Ouedraogo T, Ganame J, Fickenscher H, Kynast-Wolf G, Becher H (2006). Seroprevalence of six different viruses among pregnant women and blood donors in rural and urban Burkina Faso: a comparative analysis. J Med Virol.

[20] Tao I, Bisseye C, Nagalo BM, Sanou M, Kiba A, Surat G (2013). Screening of hepatitis G and Epstein-Barr viruses among voluntary non remunerated blood donors (VNRBD) in Burkina Faso. West Africa Mediterr J Hematol Infect Dis.

[21] Makuwa M, Mintsa-Ndong A, Souquiere S, Nkoghe D, Leroy EM, Kazanji M (2009). Prevalence and molecular diversity of hepatitis B virus and hepatitis delta virus in urban and rural populations in northern Gabon in Central Africa. J Clin Microbiol.

[22] Chotun N, Preiser W, van Rensburg CJ, Fernandez P, Theron GB, Glebe D (2017). Point-of-care screening for hepatitis B virus infection in pregnant women at an antenatal clinic: a south African experience. PLoS One.

